# The EGNOS Augmentation in Maritime Navigation †

**DOI:** 10.3390/s22030775

**Published:** 2022-01-20

**Authors:** Anna Innac, Antonio Angrisano, Silvio Del Pizzo, Giovanni Cappello, Salvatore Gaglione

**Affiliations:** 1 Department of Sciences and Technologies, University of Naples Parthenope, 80100 Naples, Italy; silvio.delpizzo@uniparthenope.it (S.D.P.); giovanni.cappello001@studenti.uniparthenope.it (G.C.); salvatore.gaglione@uniparthenope.it (S.G.); 2 Department of Engineering, Messina University, 98124 Messina, Italy; angrisanoa@unime.it

**Keywords:** maritime, EGNOS, positioning

## Abstract

The objective of this work is the evaluation of the performances of EGNOS (European Geostationary Navigation Overlay System) augmentation system in maritime navigation by comparing them with those obtained by other positioning methods as Single Point Positioning (SPP) and Differential Global Positioning System (DGPS). Preliminarily, EGNOS performances in an open-sky context were evaluated through static data downloaded by EGNOS RIMS (Ranging and Integrity Monitoring Stations) located in Rome. Then, for the maritime test carried out onboard a boat in the Gulf of Naples, two dual-frequency receivers were used: Xiaomi Mi 8 smartphone and u-blox ZED-F9P multi-band GNSS (Global Navigation Satellite System) receiver, both in kinematic mode. At last, IMO (International Maritime Organization) requirements, established in IMO Resolution A.1046 (27), that a SBAS (Satellite Based Augmentation System) system in particular scenarios (coastal, inland-water, harbor navigation and ocean waters) must respect, were verified.

## 1. Introduction

The Global Navigation Satellite System (GNSS) has a key role in different sectors, from vehicular, marine and air navigation, to “social” location-based services, using satellite positioning in non-professional applications such as in tourism or sport [1].

In the maritime sector, GNSS has become the primary way to obtain Position, Navigation, and Timing (PNT) information at sea. Since 1995, with the full operational capability of the Global Positioning System (GPS), the use of GNSS became a revolution in this field. Consequently, the maritime community is going to rapidly adopt new performance standards for shipborne GPS receiver equipment, and the International Maritime Organization (IMO) recognized GPS and GLONASS (GLObal NAvigation Satellite System) as part of the World-Wide Radio Navigation Systems (WWRNS) for ocean water navigation [2]. In 2015 and 2016 Galileo and Beidou also joined the WWRNS [3,4].

The International Maritime Organization (IMO) is an agency of the United Nations responsible for improving the safety and security of international shipping, as recalled in the Safety of Life at Sea (SOLAS) Convention of 1974 [3]. The SOLAS Convention was updated and amended through Resolutions issued by the IMO Assembly, the IMO Maritime Safety Committee (MSC), and other IMO bodies. In addition, another objective of IMO is to prevent maritime and atmospheric pollution by maritime vessels. Summarizing, all the standards used in the maritime sector for safety, security and environment are set by the IMO [5].

As recently reported by the EMSA (European Maritime Safety Agency) up to 60% of maritime navigation errors are human made; so, the people steering the ships can make mistakes that can have catastrophic consequences [6]. Accurate and reliable navigation solutions can prevent most of these accidents. In fact, today all cargo ships in the world with a capacity more than 500GT are equipped with GNSS receivers (transport requirement of the International Convention for the Safety of Life at Sea (SOLAS)). Furthermore, IMO MSC, in 2015 resolution MSC.401 [7], requires the use of at least two independent constellations of GNSS to calculate a reliable estimate of Position Velocity Time (PVT) solution. Regarding the required level of resilience and integrity in the PVT solution, MSC.401 recommends that on-board equipment is able to provide augmentation data to enhance the performance of the PVT solution in terms of accuracy, availability, continuity and integrity [8,9,10,11].

Using GNSS alone in critical maritime operations; e.g., navigating near ports, is not sufficient to carry out these operations safely. To guarantee the required performance of the PVT solution, the augmentation systems are required during these critical operations. Three types of augmentation systems (AS) for GNSS are available: Satellite Based Augmentation Systems (SBAS), Ground Based Augmentation Systems (GBAS) and Aircraft Based Augmentation System (ABAS). SBAS and GBAS provide integrity information and differential corrections by satellite and ground infrastructures, respectively. ABAS is able to obtain integrity information, using only measurements available on-board; Receiver Autonomous Integrity Monitoring (RAIM) is a common ABAS realization, only making use of redundant GNSS measurements.

The current study focuses on SBAS. The SBAS systems broadcast supplementary data to GNSS users via Geostationary Earth Orbit (GEO) satellites. SBAS consists of a network of Reference Stations (RS) covering a large area. These RSs collect data from visible GNSS satellites and further relay them to master stations, computing integrity and differential information for each visible GNSS satellite. This information is uploaded, along with the navigation message, to the GEO satellites, which transmit them to users within the coverage area [8].

SBAS is used and implemented worldwide. Similar systems are being developed by the US (Wide Area Augmentation System—WASS), Europe (European Geostationary Navigation Overlay Service—EGNOS), Japan (MTSAT Satellite based Augmentation System—MSAS), India (GAGAN), China (BD-SBAS) and Russia (System of Differential Correction and Monitoring). Thanks to these systems, the positioning characteristics of GNSS systems such as accuracy, reliability, continuity, availability and, especially, integrity can be considerably improved [8,11].

EGNOS is the European SBAS, used for augmenting GPS and GLONASS. The purpose of the system is the transmission of differential corrections and information about failures of the GNSSs via satellites located in geostationary orbits as deeply detailed in chapter II.

The maritime community is interested in EGNOS service tailored to the operational needs [12] in the European region, for improving accuracy and integrity information, especially where there is lack of infrastructure. However, even if more than 90% of maritime GNSS receiver models are SBAS compatible, only IMO MSC.401 and IEC 61108-4 (Shipborne DGPS and DGLONASS maritime radio-beacon receiver equipment) allow for the use of SBAS receiver in vessels, but there are no guidelines for their implementation. For this reason, European Commission (EC), European Space Agency (ESA) and the European Satellite Service Provider (ESSP) are working on common guidance for the manufactures for the implementation of SBAS in vessel receivers to be compliant with the operational requirements defined by the IMO.

The aim of this work is to evaluate the performance of EGNOS augmentation in maritime navigation to mitigate the undesired effects obtained when GNSS navigation is conducted in “hostile” scenarios and, consequently, to provide an alternative method able to improve the accuracy of the navigation solution. In detail, corrections provided by the EGNOS Message Server (EMS) are applied in post processing using the open-source software RTKlib [13]. The assessment analysis was conducted on several data-collections carried out both in static and kinematic modes. Static data has involved 21 h of observations downloaded by a reference station belonging to EGNOS ground segment (RIMS). While a kinematic survey has been carried out onboard a small boat collecting raw data during different navigation phases such as the unberthing, cruise and turning. The boat was equipped with two low-cost GNSS receivers: the Ublox ZED F9P and the built-in smartphone chipset Broadcom BCM47755. The positioning solutions obtained by applying EGNOS corrections have been compared to positioning solutions computed using GPS-only Single Point Positioning (SPP), code-based differential positioning modes. Finally, the performances of all results have been evaluated by considering the PPK (Post Processing Kinematic) solution as ground truth. Hence, the performance analysis was conducted in terms of accuracy, availability and continuity, also verifying the satisfaction of IMO requirements. From the results, the accuracy improvements obtained thanks to the use of EGNOS corrections are evident, especially regarding the vertical component of the position. These results highlight the importance of using EGNOS to obtain accurate estimate of vertical component of the position that is a key parameter for the analysis of the sea conditions, which starts from the survey of the amplitudes of the vessel motions [14].

The paper is organized as follows: in Section 2 a description of EGNOS architecture is provided and its role in maritime sector is illustrated, while experimental setup is described in Section 3 and results are summarized in Chapter 4. Section 5 concludes the paper.

## 2. EGNOS Background

ESA initiated the agreements for a European SBAS in 1995. The development of EGNOS started in 1999, following the agreements between ESA, the EC, Eurocontrol and national service providers air navigation. On the first of April 2009, EGNOS was handed over to the EC and starting from the first of October 2009 the corrective data were transmitted free of charge to public users [12].

EGNOS consists of a ground segment, a support segment, a space segment and a user segment. The ground segment contains 40 Ranging and Integrity Monitoring Stations (RIMS), two Mission Control Centers (MCCs) and six Navigation Land Earth Station (NLES). The communication between the components of the ground segment is guaranteed by the EGNOS Wide Area Network (EWAN). RIMS stations cover all of Europe and collect data from GNSS satellites. The Central Processing Facilities (CPF) broadcast the collected data to the MCC every second. At the MCC, the data are processed, and corrections are generated in the form of EGNOS messages. These messages are then forwarded to the NLES by the MCC’s CPF. EGNOS messages are sent to the GEO satellites by the NLES.

The support segment consists of a Performance Assessment and Checkout Facility (PACF) and the Application Specific Qualification Facility (ASQF). The support segment is controlled by the EGNOS Service Provider: the ESSP.

The space segment of EGNOS is composed by three GEO satellites that are in 36,000 km altitude circular orbits and downlink EGNOS messages to the user segment at the L1 (1575.42 MHz) frequency band [12]. EGNOS GEO satellites are Astra Ses-5 (PRN 136), Astra 5B (PRN 123) and Inmarsat 4F2 Emea (PRN 126) that are currently part of the EGNOS operational platform and are transmitting the operational Signal-In-Space (SIS) to be used by EGNOS users. From 23 March 2020 onwards, the PRN123 and PRN136 satellites are operational while the PRN126 is in test mode. Inmarsat is an initiative established by the IMO in 1979 for safer maritime navigation [12].

Finally, the user segment includes anyone with an EGNOS enabled receiver.

EGNOS provides three services: the Open Service (OS), the Commercial Data Distribution Service (CDDS) and the Safety of Life (SoL) service [15,16,17].

The OS provides EGNOS data freely to anyone in Europe with an EGNOS enabled GNSS receiver and provides a horizontal position accuracy of 1 to 3 m, and a vertical position accuracy of 2 to 4 m for GPS [17].

The SoL service is able to provide highly accurate positioning and integrity data for safety-critical application areas. This service is primarily provided for aviation, and subsequently it is also widely used in the maritime, rail and road applications sectors. Alerts are sent to the user in the event that accuracy worsens, so that the user can react to degraded navigation signals without serious consequences [16].

The CDDS is a service designed for professional users and provides EGNOS data via EGNOS Data Access Service (EDAS). This service offers raw GPS, GLONASS and EGNOS observation data to the user with an update rate of one second [15]. There are multiple EDAS services depending on the user’s application. Among them, the EDAS Signal in Space (SiS) service through the Internet (SISNet) provides the user with EGNOS messages using the Internet. These data are provided according to the SISNeT protocol, and the user must be registered to access the service. The EGNOS messages are also available for post-processing purposes via EDAS FTP. This service provides EDAS/EGNOS data in different formats and at different time intervals. A standard FTP client is used to access this service and the user must register. EDAS is available also from the network transport of RTCM (Radio Technical Commission for Maritime Services organization) via Internet Protocol (NTRIP). The data available through this service are DGNSS corrections and carrier phase measurements. This service also provides messages for Real-Time Kinematic (RTK) applications [15].

**Figure 1:**
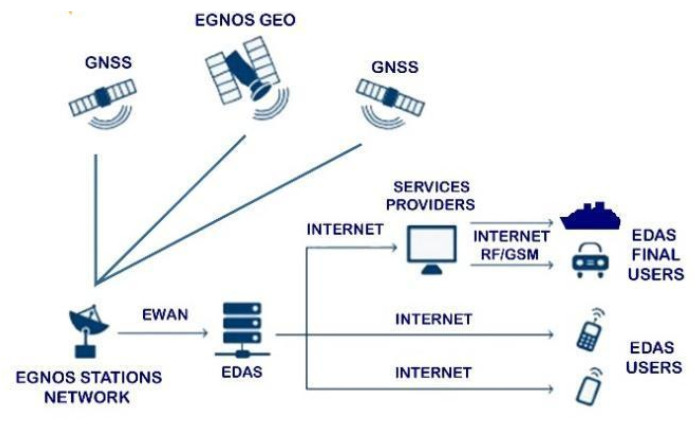
EGNOS architecture [12].

The EGNOS functional architecture is shown in Figure 1.

### EGNOS in Maritime

The ongoing initiatives for the implementation of EGNOS services for the maritime sector start from the consideration that the corrections may be available from different transmission means, such as EGNOS SiS, or through existing radio beacon stations and Automatic Identification System (AIS).

The EGNOS SiS is considered the simplest solution and the most used approach since it is based on the transmission of an EGNOS message by the GEO satellites that can be directly received and applied to the onboard vessel receiver to increase the accuracy and integrity of the navigation solution. The messages are available at 1 Hz and are transmitted at GPS L1 frequency. Corrections from EGNOS GEO satellites are available via the EDAS FTP service. This service is an EGNOS terrestrial data service for authorized users of the service [15]. These corrections are provided in EMS file format for each hour of the day. Each line in the EMS file corresponds to an EGNOS message. The EGNOS correction messages include long- and fast-term corrections. Satellite position and clock corrections are considered long-term corrections, while fast-term corrections are pseudorange corrections. Ionospheric corrections and integrity information are also provided by EMS. Once calculated, these corrections are transmitted as “differential corrections” by at least two EGNOS geostationary satellites, covering Europe.

Consequently, the service provided by EGNOS SiS fits well with marine applications, so that ships can collect these SBAS corrections from SBAS GEO satellites and apply them to obtain an improved navigation position compared to GPS standalone. In addition, the ships could benefit from the integrity provided by EGNOS, which warns users of situations that could affect positioning error.

Different studies showed the ability of EGNOS to meet the accuracy and availability requirements for general maritime navigation established in the IMO resolution A.1046 (27) [7]. The performance objectives for maritime applications are generally broken down into ocean, coastal, inland-water, harbour navigation; the IMO Resolution A.1046 (27) requirements for these four generic cases are shown in Table 1 [7].

The satisfaction of IMO requirements in maritime is deeply detailed in [8,18]. In [8], the EGNOS performance was evaluated while considering IMO Resolution A.1046 (27) requirements. The results showed a signal availability of 99.999%, a horizontal accuracy of 0.91 m at the 95th percentile, and the regions where the IMO requirements on service availability and service continuity are met. The authors concluded that EGNOS was suitable for maritime navigation in coastal and oceanic waters.

Also, in [10], a GNSS data collection campaign of 10 days along the Norwegian coast was carried out with the aim of assessing EGNOS performance at user level in the maritime domain at high latitudes in Europe. The performance obtained with the receiver has been compared with the requirements of the IMO Res. A.1046 (27), showing good agreement.

In [11], the EGNOS availability and continuity have been evaluated within the area comprised between longitudes 13° W to 19° W and latitudes 27° N to 30° N with spatial resolution of 0.1° per 0.1°. The EGNOS Maritime Service met all IMO requirements in the geographic area of latitudes more northern than 28° N and longitudes more eastern than 16° W after reaching a Signal Availability of 99.999%, a Service Availability in 99.90% of the predefined rectangular region, and 1.06 m of Horizontal Accuracy at the 95th percentile. In contrast, the Service Continuity requirement (only required for coastal waters) was met in 62.50% of the predefined region. The authors concluded that the continuity risk was the most limiting factor for expanding the EGNOS Maritime Service in the EGNOS south-west border of coverage.

The coverage problem can be also verified in other regions. Indeed, where latitudes do allow, GEO satellites are well-suited to support many users, especially shipping activity, given the need for mobility and the absence of land upon which to deploy terrestrial communications infrastructure. These communication gaps are therefore particularly relevant to the Polar Regions, where users often require near real-time delivery of information to ensure safety of life and efficient operations. In both Polar Regions, the geostationary satellites are used for many connectivity requirements, but physical limitations associated with visibility of these satellites at higher latitudes undermine their viability. Due to the location of GEO satellites directly above the equator and the curvature of the Earth, they have very low inclination ‘look-angles’ at high latitude parts of the globe. As a result, visibility of these satellites from the ground reduces to zero from latitude of approximately 70° to 79° (north and south). This restricts the number of corrections available and leads to a decrease in accuracy in the border areas of the coverage.

## 3. Experimental Setup

The objective of this work is verifying the performance of EGNOS augmentation using FTP service provided by EDAS to apply EGNOS messages (in EMS format) to GPS measurements collected in static and kinematic mode as explained in the 3.1 and 3.2 subparagraphs.

The data have been processed using the demo5 version of the RTKLIB that is an open-source version of RTKLIB optimized for low-cost receivers and for practical use. It is based on RTKLIB 2.4.3 version including additional solution features to deal with the sometimes very challenging measurements from lower cost receivers and is also optimized to work with multi-constellation solutions. RTKLIB supports standard and precise positioning algorithms with GPS, Glonass, Galileo, QZSS, BeiDou and SBAS. Different positioning modes are allowed using GNSS data for both real-time and post-processing such as single point positioning (SPP), DGPS (differential code approach), Kinematic, Static, Moving-Baseline, Fixed (carrier-phases- based relative approach), Precise Point Positioning (with different approaches).

In the current work, a solution, applying EGNOS corrections provided in the EMS file by the EDAS server, is obtained. EGNOS positioning solution has been compared to GPS-only SPP (without any EGNOS correction), DGPS, and PP-Kinematic solutions. In detail, the analyzed configurations are indicated as:

- “GPS SPP” referring to the positioning solutions obtained using only GPS observations in SPP mode, that uses only L1 frequencies; information broadcasted in the GPS navigation message have been used to compute satellite orbit and clock corrections and to reduce ionospheric delay while Saastamoinen model has been used for tropospheric correction;
- “GPS+SBAS SPP” indicates the positioning estimates obtained applying EGNOS corrections in SPP mode for satellite orbit and clock, ionospheric and tropospheric delays; also in this case, only L1 frequencies have been used;
- “DGPS” representing the position solutions computed applying dual-frequency code-based (L1 and L2 frequencies) differential positioning mode using GPS-only measurements; broadcast ionospheric information and Saastamoinen models have been used for, respectively, ionospheric and tropospheric corrections. In addition, for DGPS setup, observational corrections from the permanent stations located nearby the data collections’ places have been also applied. In detail, data from a permanent station located in Naples (NAPO) and belonging to Regione Campania network have been used for the static test, while Rome University (ROUN, Rome) station belonging to Lazio Network has been considered as base station for the kinematic one.
- “PP-Kinematic” solution is based on the carrier-phase measurements, and it provides the absolute position by solving the relative distance between the rover and a base station epoch by epoch. The relative distance is solved using the double difference (DD) measurement model, that involves both L1 and L2 frequencies and allows for eliminating the ionospheric effect and reducing the tropospheric one. As in the DGPS approach, the permanent station located in Naples (NAPO) was chosen as base station and measurements from GPS and Glonass systems were processed.

Finally, as it will be deeply described in Section 4, the performance of the considered configurations has been evaluated in terms of continuity, availability and accuracy in position domain. For the latter one, a positioning error analysis has been carried out considering the well-known coordinates of the selected reference station for the static test as described in subparagraph 3.1, while a reference solution has been computed for the kinematic data collection as detailed in Section 3.2.

### 3.1. Static Test

**Figure 2:**
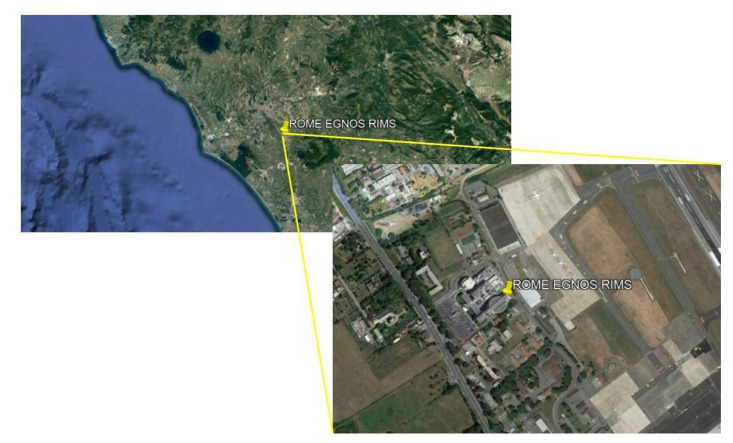
EGNOS RIMS located in the airport of Rome “Ciampino”.

Static data for 21 h, with a sample rate of 30 s, have been downloaded by EGNOS RIMS located in Rome for the day 6 November 2021. The EGNOS RIMSs can be considered as static reference receivers, which are placed at fixed and known surveyed locations whose coordinates can be used as reference for the positioning error analysis. The position of the considered station is shown in Figure 2.

**Figure 3:**
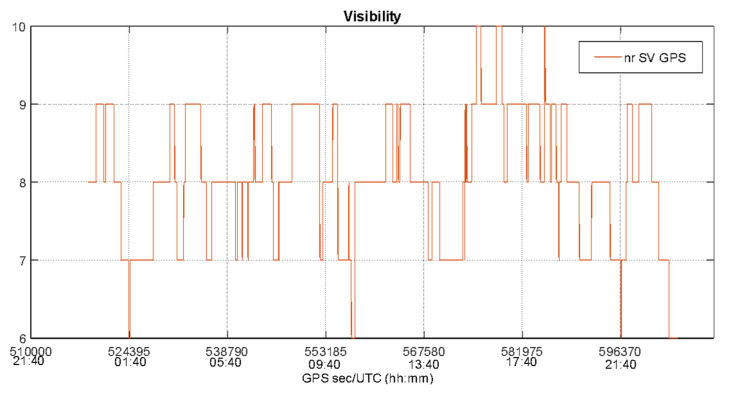
Number of visible GPS satellites during the static test.

**Figure 4:**
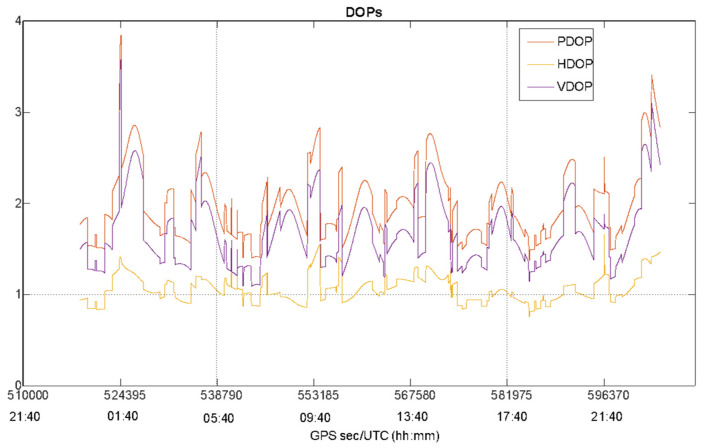
Position Dilution of Precision (PDOP), Vertical DOP (VDOP) and Horizontal DOP (HDOP)values as function of the time.

The static test has been considered to carry out a preliminary evaluation of EGNOS performance in an open-sky scenario characterized by good satellite visibility and geometry as confirmed by Figure 3 that plots the number of GPS satellites as function of time, while Figure 4 shows the behavior of Position Dilution of Precision (PDOP), Vertical DOP (VDOP) and Horizontal DOP (HDOP) during the whole test.

The Figure 3 and Figure 4 confirm the good satellite visibility and geometry of the operational scenario. The number of GPS satellites varies from a minimum of six satellites to a maximum of ten, with an average of eight visible GPS satellites also guaranteeing a solution availability and continuity equal to 100%. In Figure 4, the same behavior can be noted for the PDOP, VDOP and HDOP curves, with lower values for HDOP: the DOP values do not exceed the threshold of 3 (with the exception of some epochs at the beginning and end of the data collection) indicating a good satellite geometry for the entire duration of the test.

### 3.2. Kinematic Test

**Figure 5:**
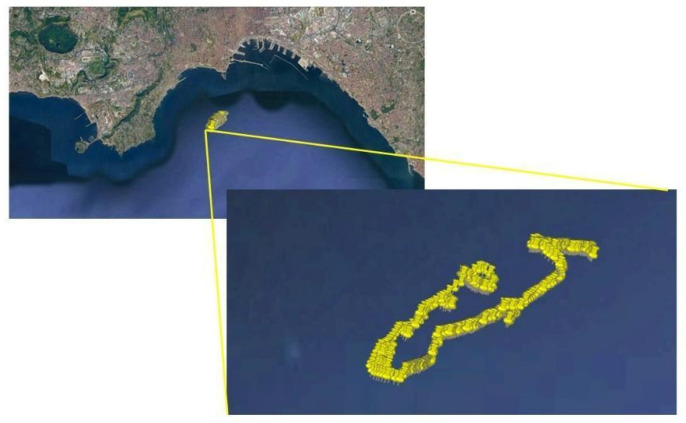
Trajectory travelled by the boat in the Gulf on Naples on 29 September 2021.

On 29 September 2021, a kinematic data collection was carried out onboard a small boat, during an oceanographic campaign in the Gulf of Naples as shown by the trajectory in the Figure 5. One hour of 1 Hz measurements was collected using a smartphone and a GNSS receiver located approximately at the center of mass of the boat. In detail, two sensors were used in the kinematic test to collect the raw data:

- Xiaomi Mi 8, an Android smartphone equipped with a Broadcom BCM47755 chipset that is a dual-frequency GNSS chip able to track navigation messages, PR observables, accumulated delta ranges for GPS, Glonass, BeiDou and Galileo. The application used to store the raw observables in a RINEX (Receiver Independent Exchange Format) file is Rinex ON application;
- u-blox ZED-F9P, a multi-band GNSS receiver able to the concurrent reception of GPS, GLONASS, Galileo and BeiDou. The device was connected to a multi-band GNSS patch antenna capable of receiving L1 and L2 frequencies [19] and the software dedicated to collection of the raw data.

**Figure 6:**
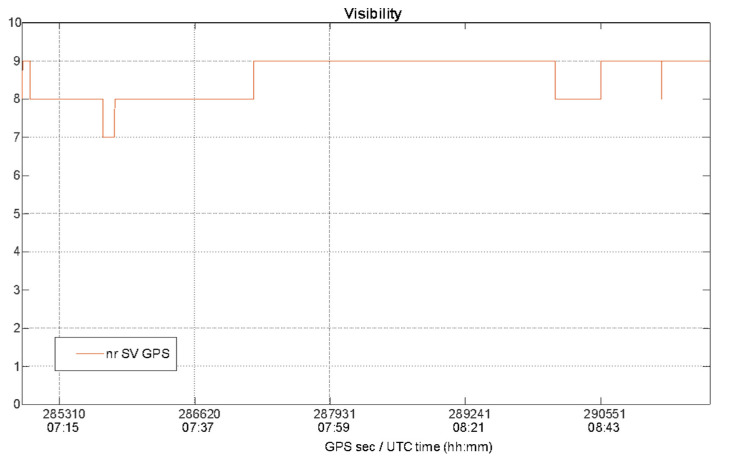
Number of visible GPS satellites during the kinematic test.

**Figure 7:**
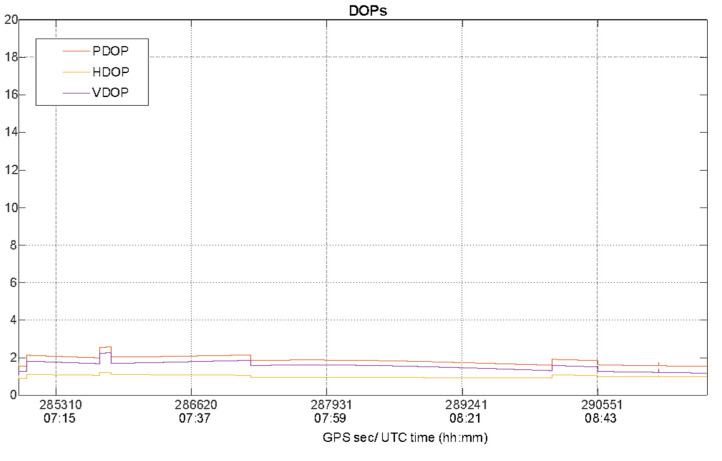
Position Dilution of Precision (PDOP), Vertical DOP (VDOP) and Horizontal DOP (HDOP)values as function of the time during the kinematic test.

Especially for the kinematic data collection mode, the satellite visibility and geometry must be investigated, since the boat is moving and, consequently, the characteristics of the operational context change epoch by epoch. For this analysis, data collected by the u-blox were used. The number of GPS satellites is plotted as function of time in Figure 6, while the PDOP, VDOP and HDOP values during the kinematic test are shown in Figure 7.

During the kinematic test the maximum percentage of solution availability and continuity was guaranteed for all the duration of the test as confirmed by satellite visibility shown in the Figure 6 where the number of satellites varies from 7 to 9 with a mean equal to 8.6, showing a good satellite visibility as confirmed by the behavior of satellite geometry in terms of higher DOP values shown in Figure 7.

**Figure 8:**
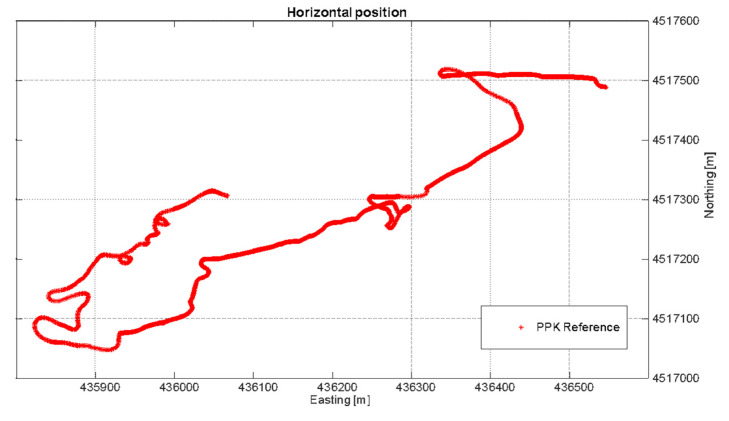
The horizontal positioning solutions obtained applying PPK mode and used to perform the error analysis in the kinematic test.

**Figure 9:**
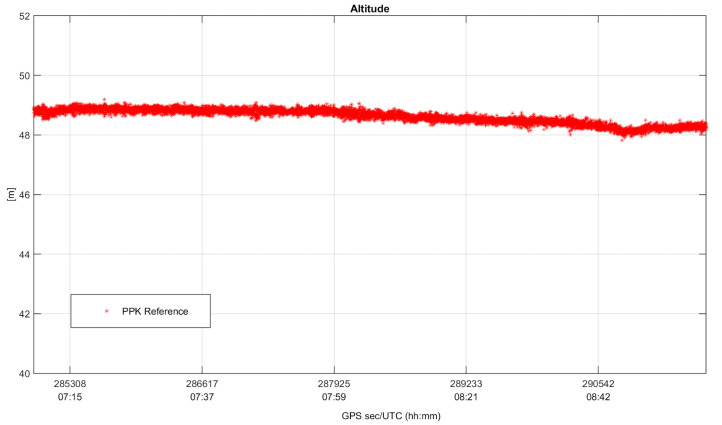
The altitude values obtained applying PPK mode and used for the error analysis.

Since the test is kinematic, in order to perform an error analysis in the position domain, it was necessary to generate a ground truth. The reference trajectory is obtained processing the raw data collected by the u-blox receiver in a carrier-based Kinematic positioning (PPK) method (performed by RTKlib as well) and using the permanent station NAPO as base. The PPK approach is widely used for determining position solutions in high accuracy, and such a condition is guaranteed by fixing the carrier phase ambiguity to their theoretical integer values. In Figure 8 and Figure 9, horizontal positioning solutions and altitude values obtained using PPK mode for the whole duration of the test are shown, considered as ground truth for the analysis conducted in Section 4.2.

## 4. Results and Discussion

After a preliminary analysis of the operational scenarios, raw data collected in static and kinematic modes were processed using the different setups described in Section 3 such as GPS SPP, GPS+SBAS SPP and DGPS configurations. The aim was to analyze the performance of EGNOS augmentation in terms of solution accuracy, availability and continuity.

For the performance analysis in terms of accuracy, positioning solutions obtained using the three configurations have been compared to reference ones computing vertical and horizontal positioning errors. The figures of merit adopted for this analysis are Root Mean Square (RMSE), mean and maximum errors for both horizontal (H), vertical (Up) and tridimensional (3D) components of position. Furthermore, the capability to meet accuracy requirements as defined by IMO about horizontal accuracy of 10 m was also checked.

In addition, the performance of the different configurations was also verified in terms of solution availability (computed as the time percentage when the estimated solution is simply available, i.e., the number of available satellites is sufficient for the positioning) and continuity (considered as the probability that the positioning solution is available over a temporal period of 15 min).

### 4.1. Static Test

Table 2 summarizes the figures of merit adopted for performance analysis of positioning solutions obtained by processing static data downloaded by the selected EGNOS RIMS (Section 3.1) and applying GPS SPP, GPS + SBAS SPP and DGPS setups.

From Table 2, it can be noted that EGNOS corrections provide the best performance in terms of all considered figures of merit, with an accuracy improvement of meter order especially on vertical component. In particular, horizontal and vertical mean error are, respectively, equal to 1.1 and 1.9 m for GPS SPP and to 1.3 and 1.6 m for DGPS, while they are equal to 0.5 and 0.8 m after applying EGNOS corrections. Furthermore, comparing GPS SPP and DGPS, even if GPS SPP shows better performance on horizontal and vertical planes, DGPS performs lower values for tridimensional accuracy. The same trend can be noted both for RMS and maximum errors, as well as for the position components. Furthermore, the horizontal errors are always less than 10 m, fully satisfying the IMO requirement for horizontal accuracy. Finally, as discussed in Section 3.1, IMO requirements such as signal availability and continuity are fully satisfied as well: indeed, they are equal to 100%, as shown in Table 2.

**Figure 10:**
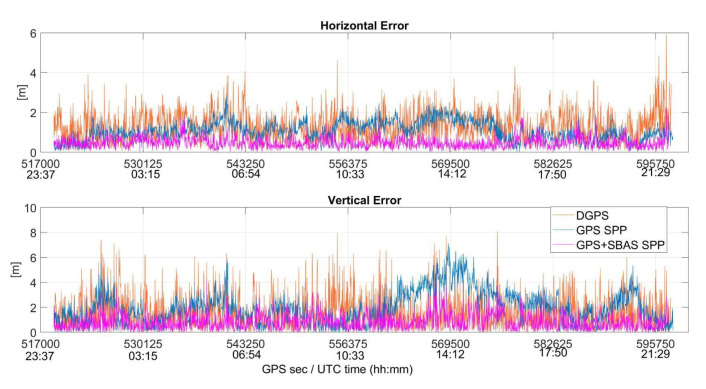
Horizontal and vertical positioning errors (in meters) as function of the time for GPS SPP (blue line), GPS + SBAS SPP (magenta line) and DGPS (orange line) configurations.

Horizontal and vertical position errors for the different configurations are plotted as function of time in Figure 10 where blue, magenta and orange lines are relative to GPS SPP, GPS + SBAS SPP and DGPS configurations, respectively.

It is evident that the use of information contained in EMS file allows for having a solution more stable than the other configurations GPS + SBAS SPP, as shown in Figure 10. Furthermore, the figure shows the improvement obtained on a vertical component, with GPS + SBAS SPP vertical errors lower than others.

### 4.2. Kinematic Test

As detailed in Section 3.2, raw data have been collected in kinematic using the u-blox receiver and the smartphone Xiaomi Mi 8 located onboard a small boat. The data from both devices has been processed using the considered configurations (described in 3), except for IF combination used for the ionospheric correction in DGPS setup using data from the Xiaomi smartphone due to the absence of dual frequency measurements for some GPS satellites. Finally, the positioning solutions obtained through processing the data from the smartphone have been compared to the reference solution described in 3.2.

Table 3 shows the performance analysis of positioning solutions obtained processing data collected by the u-blox receiver and the Xiaomi Mi 8 devices.

Using the u-blox device, the best performance in terms of accuracy on vertical component of the position can be highlighted for GPS + SBAS SPP configuration, with differences of meter order, especially with respect to GPS SPP. On the other hand, DGPS shows comparable performance on a vertical plane, even with differences of decimeter order compared to the other configurations, while worsening behavior on a horizontal plane can be observed for DGPS. In fact, mean and RMS values on a horizontal plane vary from 0.99 and 1.01 m (with a maximum error value equal to 2.00 m) for DGPS, to 0.68 and 0.72 m for GPS + SBAS SPP (and a maximum error equal to 1.89 m). Furthermore, also in this case, as static one, the horizontal errors are always less than 10 m, fully satisfying the IMO requirement for horizontal accuracy. Finally, as discussed in Section 3.2, IMO requirements such as signal availability and continuity are fully satisfied: indeed, they are equal to 100%, as can be noted in Table 3. Focusing on horizontal accuracy, it is possible to notice that the DGPS solution is worse than the GPS SPP solution and better than on vertical accuracy. Despite this, the 3D accuracy of DGPS is better than GPS SPP.

Conversely, analyzing the results obtained processing data collected by the Xiaomi Mi 8, the figures of merit considered for the performance analysis in terms of positioning accuracy are higher compared to u-blox ones, as can be noted in Table 3. In this case, the best performance can be noted for a GPS SPP configuration on horizontal component of the position, while a DGPS configuration provides lower values than GPS SPP on a vertical plane. Indeed, a GPS + SBAS SPP configuration fails at giving worse performance compared to the others. Furthermore, all the configurations provide horizontal errors lower than 10m, with probability values higher than 99%. Also, in this case, the DGPS solution is characterized by a worse horizontal accuracy than GPS SPP and a better vertical accuracy; however, as previously highlighted, 3D accuracy is always better than GPS SPP. Finally, the performance analysis in terms of signal availability and continuity obtained using the GPS SPP configuration to process data from the smartphone satisfies the thresholds defined by IMO, and conversely to the GPS + SBAS and DGPS.

**Figure 11:**
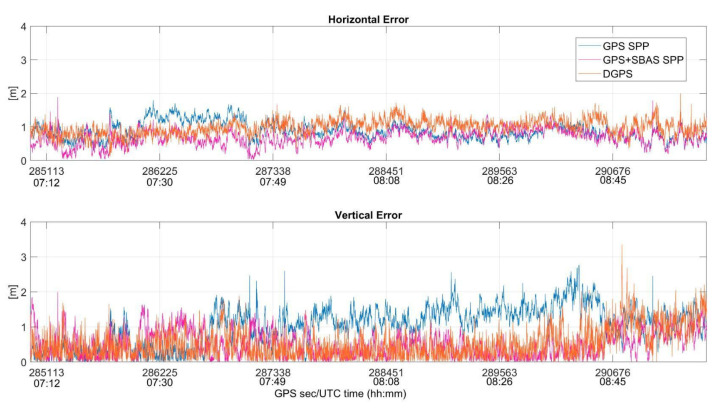
Horizontal and vertical positioning errors (in meters) as function of the time for GPS SPP (blue line), GPS + SBAS SPP (magenta line) and DGPS (orange line) configurations applied to data from u-blox receiver.

**Figure 12:**
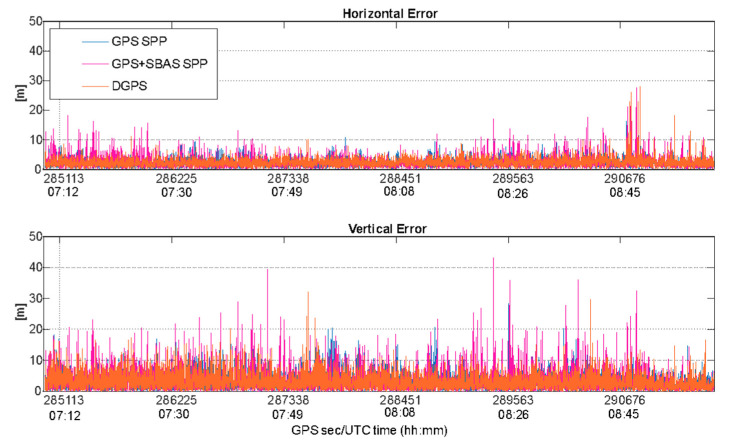
Horizontal and vertical positioning errors (in meters) as function of the time for GPS SPP (blue line), GPS + SBAS SPP (magenta line) and DGPS (orange line) configurations applied to data from Xiaomi Mi 8.

Indeed, the behaviors of horizontal and vertical errors obtained by applying the three configurations to data collected by the u-blox and Xiaomi Mi 8 devices are also shown, respectively, in Figure 11 and Figure 12.

Both figures confirm the results summarized in Table 3. In detail, Figure 11 shows the best performance obtained thanks to the use of EGNOS corrections, especially on vertical components of the position. Conversely, using data from the smartphone, the configurations perform similarly, as can be noted in Figure 12, with a smaller enhancement obtained using the GPS SPP setup, especially on a horizontal plane. However, the errors obtained using data from the smartphone are higher than the ones shown in Figure 11, which is related to the characteristic of smartphone measurements that are noisier than a usual mass market receiver, as is well known [14,20].

## 5. Conclusions

Most GNSS receivers are capable of receiving and processing EGNOS signals and can be used to support numerous applications, especially in the maritime environment. The choice of SBAS/EGNOS enabled receivers leads to obtaining better position performance; this is directly correlated with an improvement in navigation safety and an enhancement of those services based on PNT information.

In the present work, to verify the performance EGNOS, static and kinematic data collections have been processed using EGNOS corrections available on an EDAS server. According to the presented experiments, the use of EGNOS corrections provides a more accurate positioning solution in respect to using only GPS measurements in both SPP and DGPS positioning modes. As summarized in Table 2 and Table 3, evident enhancements on vertical and horizontal components of the position are provided by GPS + SBAS SPP configuration. This latter configuration also outperforms DGPS, except when measurements collected by a smartphone have been used.

Furthermore, although there are no performance requirements on the vertical component (as defined by IMO for the horizontal one) despite its importance in different applications, this analysis was investigated in the present study. In fact, the significant enhancement obtained on a vertical component is particularly interesting for applications requiring accurate estimate of the altitude like the determination of the sea state conditions, encountered by the ship in the course of its route, starting from the survey and analysis of vessel motions [14,21]. Indeed, the real-time knowledge of the sea spectrum is of fundamental importance for different aspects ranging from the safety of navigation to comfort on board. The obtained results highlight improvements that can be provided by EGNOS corrections for several real maritime applications. In addition, these results can represent a start point for further analysis about EGNOS services that can be suitable for maritime application, and, for example, integrity monitoring.

## Figures and Tables

**Table 1:** Requirements for a general maritime navigation established in the IMO Resolution A.1046 (27) [5,8].

Parameters	Navigation Scenario	
	Coastal, Inland-Water, Harbour Navigation	Ocean Waters
Horizontal Accuracy 95%	10 m	100 m
Signal Availability	99.8%	99.8%
Continuity (over 15 min)	99.97%	NA
Position update rate	2 s	2 s
Time to Alarm	10 s	As soon as practical by Maritime safety information
Coverage	Local	Global

**Table 2:** Summary results of performance analysis conducted in terms of accuracy (using as figures of merit Root Mean Square (RMS), mean and maximum errors) for both horizontal (H) and vertical (Up) components of position, tridimensional accuracy (using as figures of merit Root Mean Square (RMS) and mean errors), IMO parameter on absolute horizontal accuracy, availability and continuity (over 15 min).

Conf.	Horizontal Accuracy [m]				Vertical Accuracy [m]			3D Accuracy [m]		Continuity (15 min) (%)	Signal Avail. (%)
	Mean	RMS	Max	Req. Imo 10 m (%)	Mean	RMS	Max	Mean	RMS		
GPS SPP	1.07	1.17	2.96	100	1.94	2.34	7.14	2.32	2.62	100	100
GPS + SBAS SPP	0.49	0.56	2.25		0.84	1.06	4.24	1.04	1.20		
DGPS	1.32	1.51	5.94		1.64	2.11	8.07	2.29	2.59		

**Table 3:** Summary results of performance analysis conducted in terms of accuracy (using as figures of merit Root Mean Square (RMSE), mean and maximum errors) for both horizontal (H) and vertical (Up) components of position, tridimensional accuracy (using as figures of merit Root Mean Square (RMS) and mean errors), IMO parameter on absolute horizontal accuracy, availability and continuity (over 15 min) using data collected in kinematic mode by the u-blox receiver and the Xiaomi Mi 8 devices.

Device	Conf.	Horizontal Accuracy [m]				Vertical Accuracy [m]			3D Accuracy [m]		Contin. (15 min) (%)	Signal Avail. (%)
		Mean	RMS	Max	Req. Imo 10 m (%)	Mean	RMS	Max	Mean	RMS		
u-blox	GPS SPP	0.88	0.92	1.80	100	1.03	1.17	2.77	1.43	1.49	100	100
	GPS + SBAS SPP	0.68	0.72	1.89		0.50	0.61	1.99	0.90	0.94		
	DGPS	0.99	1.01	2.00		0.52	0.67	3.35	1.18	1.22		
Xiaomi Mi 8	GPS SPP	2.30	2.70	16.31	99.9	3.40	4.43	28.31	4.42	5.19	100	100
	GPS + SBAS SPP	2.42	3.06	27.66	99.0	3.67	5.09	43.17	4.71	5.94	100	97.8
	DGPS	2.44	2.87	28.01	99.6	3.26	4.25	32.12	4.41	5.13	99.73	99.9
